# Habitat Loss and Other Threats to the Survival of *Parnassius apollo* (Linnaeus, 1758) in Serbia

**DOI:** 10.3390/insects16080805

**Published:** 2025-08-04

**Authors:** Dejan V. Stojanović, Vladimir Višacki, Dragana Ranđelović, Jelena Ivetić, Saša Orlović

**Affiliations:** 1 Institute of Lowland Forestry and Environment (ILFE), University of Novi Sad, Antona Čehova 13d, 21102 Novi Sad, Serbia; dejanstojanovic021@yahoo.co.uk (D.V.S.); sasao@uns.ac.rs (S.O.); 2 Institute for Technology of Nuclear and Other Mineral Raw Materials, Franše d’ Eperea 86, 11000 Belgrade, Serbia; d.randjelovic@itnms.ac.rs; 3 Faculty of Technical Sciences, University of Novi Sad, Trg Dositeja Obradovića 6, 21000 Novi Sad, Serbia; jelenaivetic@uns.ac.rs

**Keywords:** *Parnassius apollo*, habitat succession, traditional grazing, *Sedum album*, vegetation indices, climate stress, wildfire impact

## Abstract

The cessation of traditional mountain grazing has emerged as a principal driver of habitat degradation and the local extinction of *Parnassius apollo* (Linnaeus, 1758) in Serbia. While previous studies have cited multiple contributing factors, our research provides evidence that the abandonment of extensive livestock grazing has triggered vegetation succession, the disappearance of the larval host plant (*Sedum album*), and a reduction in microhabitat heterogeneity—conditions essential for the persistence of this stenophagous butterfly species. Through satellite-based analysis of vegetation dynamics (2015–2024), we identified clear structural differences between habitats that currently support populations and those where the species is no longer present. Occupied sites were characterized by low levels of exposed soil, moderate grass coverage, and consistently high shrub and tree density, whereas unoccupied sites exhibited dense encroachment of grasses and woody vegetation, leading to structural instability. Furthermore, MODIS-derived indices (2010–2024) revealed a consistent decline in vegetation productivity (GPP, FPAR, LAI) in succession-affected areas, alongside significant correlations between elevated land surface temperatures (LST), thermal stress (TCI), and reduced photosynthetic capacity. A wildfire event on Mount Stol in 2024 further exacerbated habitat degradation, as confirmed by remote sensing indices (BAI, NBR, NBR2), which documented extensive burn scars and post-fire vegetation loss. Collectively, these findings indicate that the decline of *P. apollo* is driven not only by ecological succession and climatic stressors, but also by the abandonment of land-use practices that historically maintained suitable habitat conditions. Our results underscore the necessity of restoring traditional grazing regimes and integrating ecological, climatic, and landscape management approaches to prevent further biodiversity loss in montane environments.

## Simple Summary

The Apollo butterfly (*Parnassius apollo*) is one of the most striking yet critically threatened butterfly species in Europe. In Serbia, it has vanished from many sites where it historically occurred. This study identifies the primary cause of local extinction as the abandonment of traditional livestock grazing, particularly by sheep and goats, which historically maintained open, sunlit mountain meadows. In the absence of grazing, these habitats undergo ecological succession, becoming dominated by tall vegetation and shrubs, rendering them unsuitable for the survival of this species. Additionally, climate warming and a severe wildfire on Mount Stol in 2024 have further degraded critical habitats. By combining satellite-based remote sensing with field surveys, we compared areas where the species persists with those where it has disappeared. Our findings highlight the urgent need to reintroduce traditional grazing practices and preserve open mountain grasslands to support the recovery and long-term conservation of *P. apollo* in Serbia.

## 1. Introduction

### 1.1. Taxonomic Background and Regional Status

*Parnassius apollo* (Linnaeus, 1758), stands as one of the most iconic butterflies of Eurasian montane ecosystems, first described from southern Sweden. Over the decades, more than 200 subspecies have been proposed across its expansive range, especially in the Alps. However, recent taxonomic revisions have cast doubt on the validity of many of these forms, suggesting that a substantial number were established largely for commercial purposes—to artificially enhance specimen value and stimulate commercial collection and trade [1]. It is a charismatic butterfly species often associated with alpine and subalpine habitats [2], particularly in Western Europe. However, across much of its range in Central and Eastern Europe, as well as parts of Asia, the species occurs at lower elevations, inhabiting open, rocky calcareous slopes and limestone outcrops, often in warm, xeric conditions [3,4]. In these areas, it is not restricted to high-altitude environments but thrives in habitats shaped by traditional land use, such as moderate grazing and haymaking, which prevent forest encroachment and maintain open conditions suitable for its larval food plants (e.g., *Sedum* spp.)

As a stenophagous species restricted to ecologically narrow alpine and subalpine habitats, *P. apollo* is acutely vulnerable to climatic shifts and habitat degradation [1]. Throughout Europe, its populations have experienced precipitous declines, with confirmed local extinctions in several Central European mountain systems [1]. In Finland, population collapses began as early as the 1930s, leading to disappearance from numerous formerly occupied sites within three decades—a pattern attributed primarily to increased atmospheric deposition of heavy metals linked to industrial emissions [5,6].

In Serbia, *P. apollo* has been documented for over a century [7,8], with two recognized subspecies: *jelicus Fruhstorfer* and *timacus Varga & Lelo* [9]. In the eastern region of Timočka Krajina, three historical strongholds have been identified—Stol, Janošica, and Vetren. While only sporadic individuals have been reported at Janošica and Vetren, the population at Mount Stol—proposed as a geographically distinct and taxonomically valid subspecies (*timacus* ssp. nov.)—was once estimated at several hundred individuals [8]. The Stol habitat is defined by its limestone substrate and the presence of *Sedum album*, the larval host plant, with notable year-to-year fluctuations in population size recorded during the late 1960s and early 1970s.

Approximately 70 km southeast of Mount Stol, near the Bulgarian border, four additional historical sites have been noted: Janošica and Vetren (as above), and Topli Do (800 m) and Toplodolska Reka (900 m) [10]. These localities represent the nearest historical populations to Stol. Recent findings from Stara Planina [11] confirm the continued presence of the species and its protection under several international conservation frameworks, including the EU Habitats Directive (Annex IV), CITES, and the European Red List of Butterflies, where it is classified as Near Threatened (NT) [12]. In Serbia, it is listed as Vulnerable (V) according to national IUCN criteria [10].

Additional records exist from Mount Kopaonik, at two sites: Bele Stene and Đorov Most [13,14]. However, no viable population has been observed at Mount Stol since 2017, and Bele Stene has yielded no sightings for over seven years. These alarming regional declines prompted the present study, which includes specimens from the surviving Đorov Most population. Recently published data also confirm the occurrence of *P. apollo* at the Rastište locality on Mount Tara, near the border with Bosnia and Herzegovina [15]. In stark contrast, the species is now classified as extinct (EX) in Romania, with no confirmed observations at any of its six historical regional sites since the mid-20th [16].

The conservation crisis of *P. apollo* mirrors the broader vulnerability of emblematic insect species. As demonstrated by recent work on the monarch butterfly (*Danaus plexippus*), strong public sentiment and symbolic identification with charismatic taxa can substantially boost conservation engagement and funding [17]. These insights underscore the potential of *P. apollo* as a flagship species for safeguarding high-altitude grassland ecosystems in Southeastern Europe.

### 1.2. Host Plant: Heavy Metals, Habitat, and Climatic Conditions

*Sedum album* serves as the primary larval host plant for many central and southern European populations of *Parnassius apollo* (Lepidoptera: Papilionidae), a threatened butterfly species undergoing a steep population decline. Experimental studies on the *S. album–P. apollo* interaction have revealed that elevated concentrations of secondary metabolites in the host plant can increase larval detoxification costs and impair nutrient assimilation, ultimately resulting in higher larval mortality [18,19].

*Sedum album* L. is a perennial succulent in the Crassulaceae family, distributed widely across temperate Europe—from North Africa to Scandinavia, and from the Iberian Peninsula to the Caucasus [20]. As a chamaephyte, it is one of the most frequently encountered *Sedum* species on the Balkan Peninsula, thriving in dry grasslands, rocky outcrops, and forest edges, particularly on limestone and serpentine soils [21].

Several congeners of *S. album* have been documented as efficient accumulators of heavy metals. These include *S. plumbizincicola* a Cd and Zn hyperaccumulator; [22], *S. alfredii* (Cd hyperaccumulator; [23]), *S. spectabile* (Cd accumulator; [24]), and *S. sediforme* Cu-tolerant; [25]. However, the metal accumulation potential of *S. album* under natural field conditions remains largely unexplored.

Globally, anthropogenic activities such as mining have elevated heavy metal concentrations in ecosystems, translocating metals from deep lithospheric sources into previously uncontaminated biomes [6]. Acidic precipitation enhances this impact by solubilizing metal ions and increasing their bioavailability [6,26,27].

Mining operations in proximity to Mount Stol and Mount Kopaonik have been associated with increased atmospheric deposition of metal-laden dust—an insidious yet impactful mode of metal pollution. Notably, the recent recolonization and range expansion of *P. apollo* in certain areas of Western Europe have been correlated with a regional decline in airborne metal fallout [6]. Metal content analyses of larval and host plant samples from recolonized islands in the Finnish archipelago (Kemio–Hankoniemi) suggest that the decline in heavy metal deposition in recent years may have enabled partial recovery, while elevated metal loads in the past likely contributed to historical population collapses *P. apollo* populations [6].

A broad array of laboratory and field studies has documented the deleterious effects of toxic metals on arthropods, including elevated mortality and reduced reproductive success across numerous taxa [6,28,29,30,31,32,33,34,35,36]. The highest sensitivity is generally observed in detritivores, fungivores, and parasitoids, whereas herbivores—including butterfly larvae—often exhibit comparatively lower direct mortality [37,38,39,40,41]. This relative tolerance may arise from indirect ecological effects: host plants compromised by pollution may become more susceptible to herbivory, while declines in parasitoid populations reduce mortality pressures on larval insects [6,29,37,38,39,40,41,42,43,44,45,46,47,48,49,50].

Despite multiple conservation efforts, *P. apollo* remains endangered. A notable example is the reintroduction program in Poland’s Pieniny National Park, which began in the late 20th century with a founding population of only 20–30 individuals [51]. Although initial results were encouraging, the program encountered unforeseen developmental anomalies in released butterfly individuals, including wing malformations and abnormal behavior. Subsequent analyses detected the presence of pathogenic bacteria, including *Yersinia pseudotuberculosis* (Pfeiffer) Smith & Thal and *Serratia* spp., in both normal and malformed individuals—potentially compromising long-term population viability [51].

In addition to biotic stressors, climatic conditions critically influence habitat suitability for *P. apollo*. Species distribution models show that colder climatic zones tend to favor both the butterfly and its host plants, whereas warming trends are associated with population decline and habitat contraction [52,53]. At lower elevations, abandonment of traditional land-use practices has facilitated forest encroachment, compounding the adverse effects of climate change [52]. In Serbia, the disappearance of *P. apollo* has coincided with increasingly mild winters and a marked reduction in livestock grazing—particularly by sheep, goats, and cattle. This land-use shift has led to vegetation succession, habitat homogenization, and ultimately, the degradation of once-viable butterfly habitats [52,54].

### 1.3. Ecological Pressures: Habitat Loss, Succession, and Land-Use Change

*P. apollo* is among the most visually distinctive and ecologically significant species of diurnal *Lepidoptera* in Europe. A stenophagous relict of glacial fauna, it is strictly associated with subalpine and montane ecosystems where host plants of the genus *Sedum*—particularly *S. album* and *S. telephium*—persist in sun-exposed, sparsely vegetated habitats crucial for larval development [55,56]. Yet across its range, including in Serbia, *P. apollo* has experienced precipitous declines over the past several decades.

The principal drivers of this decline are habitat loss and fragmentation, largely resulting from urban expansion, infrastructure development, intensified agriculture, and the widespread abandonment of traditional pastoral systems. These pressures are compounded by climate change, the spread of invasive vegetation, and direct anthropogenic threats such as overcollection. Notably, the cessation of seasonal grazing—a cornerstone of traditional land use across much of *P. apollo*’s range—has emerged as a critical but underappreciated cause of habitat degradation. For centuries, extensive livestock grazing shaped open, thermophilic microhabitats, maintaining the low vegetation cover required for *Sedum* proliferation and the butterfly’s reproductive success.

Following grazing abandonment, several ecologically detrimental changes occur [52,54,55,56]:

- Vegetation succession leads to the encroachment of tall grasses, shrubs, and invasive species, which block sunlight at ground level and displace sun-loving succulents like *S. album*.
- Loss of habitat heterogeneity erodes the mosaic of rocky surfaces, bare patches, and low grasses, disrupting the availability of microhabitats required for larval and adult stages.
- Decline in spatial connectivity restricts dispersal and gene flow, isolating populations and increasing vulnerability to local extinction.

These transitions typically unfold within one to two decades and, while the resulting vegetation may appear verdant, such environments become functionally depauperate and unsuitable for stenophagous taxa such as *P. apollo*.

Beyond its aesthetic appeal, *P. apollo* functions as an ecological sentinel of high-altitude grasslands. The species displays a trophic dichotomy: “albophagous” populations depend on *Sedum album*, while “telephiophagous” populations utilize *S. telephium*. This divergence is not only trophic but also historical and altitudinal, reflecting evolutionary dynamics driven by glacial-interglacial cycles [18,57]. With more than 200 described subspecies radiating from a Neogene origin [18], *P. apollo* populations have nevertheless exhibited steady declines since the 19th century—driven by a convergence of climatic shifts, anthropogenic habitat transformation, and intra-populational vulnerabilities such as genetic erosion and maladaptive behaviors [57].

While host plant availability has traditionally been viewed as a key determinant of larval presence, recent long-term studies in Finland challenge this assumption. In the southern Finnish archipelago, *Hylotelephium telephium* remained abundant, yet larval presence declined dramatically—from 75% to 20% of surveyed islands over two decades [55]. The decline was not due to host plant scarcity but rather to habitat succession, the loss of structural heterogeneity, and the abandonment of management practices like rotational grazing and shrub clearance. These processes led to the closure of formerly suitable open habitats.

Additional stressors—most notably intensified climate change—further threaten *P. apollo* metapopulations by inducing extreme weather events, shifting phenological cues, and modifying species interactions [55,56]. A recent comparative habitat analysis in southwestern Finland revealed a strong positive correlation between butterfly presence and rocky terrain rich in larval host plants and nearby nectar sources, while tree and shrub cover (a proxy for succession) correlated negatively [56]. Such results underscore the importance of maintaining complex habitat mosaics and long-range connectivity as pillars of conservation strategy.

Given the high degree of fragmentation and the extinction of numerous local populations, *P. apollo* conservation demands a multifaceted approach—combining in situ restoration, ex situ reinforcement, and the revitalization of traditional land use [56,58]. Species Distribution Models (SDMs) indicate that colder climatic phases, such as the Last Glacial Maximum (~22,000 years BP), expanded the species’ ecological niche, while interglacial warming—as seen today—has led to sharp contractions [52]. Notably, when *Sedum album* and *Hylotelephium telephium* were included in these models, the estimated suitable range for *P. apollo* narrowed considerably. This result likely stems from differing climatic tolerances between the butterfly and its host plants and supports the hypothesis that contemporary habitat loss and land abandonment exert stronger constraints than temperature shifts alone [52].

### 1.4. Conservation Challenges and Genetic Perspectives

A recent study in the Sierra Nevada revealed that populations of *Parnassius apollo* are highly spatially fragmented, with limited gene flow between the western and eastern sectors of the mountain range. Notably, the formerly occupied Puerto de la Ragua pass has been entirely depopulated, most likely due to a decline in host and nectar plant availability at lower elevations [59]. This case underscores the crucial role of microhabitat continuity in sustaining viable metapopulations.

In Poland’s Pieniny National Park, reintroduction efforts also highlight key conservation dilemmas. Excessive supplementation using captive-bred individuals—without parallel habitat restoration—resulted in a reduced carrying capacity and disrupted natural population dynamics [60]. This study emphasized the risks of poorly coordinated reintroduction initiatives and the need for integrated ecological planning to avoid so-called “overload effects.”

A geometric morphometric analysis involving 20 *P. apollo* subspecies across the Carpathian-Pannonian region found negligible morphological divergence. The authors recommend treating these lineages as a single conservation unit pending further genetic resolution [61], potentially streamlining resource allocation and management strategies. In parallel, mark–release–recapture experiments have shown that variation in terrain and vegetation structure significantly influences capture success and estimates of population size [62], suggesting that microtopographic features should be integrated into future monitoring protocols.

Although once widespread, *P. apollo* is now classified as endangered, its survival jeopardized by a convergence of genetic, ecological, and climatic threats. While reintroduction projects have been met with partial success, novel challenges have emerged, such as a higher incidence of wing malformations among semi-captive individuals. Recent research has implicated several underlying causes: mutations in the *wingless* gene, disruption of key enzymes like laccase 1 and 2, absence of *Wolbachia* symbionts, and the presence of pathogenic bacteria including *Yersinia pseudotuberculosis* and *Serratia* spp. [63].

On a broader scale, macroevolutionary studies on the genus *Parnassius* in the Himalayan–Tibetan region indicate that diversification is driven not by clade age or speciation rate, but by ecological opportunity—i.e., niche saturation in isolated mountain environments [64]. Supporting this, a recent reference genome for *P. apollo*—among the most complete for Lepidoptera—has revealed a highly conserved chromosomal structure and notable genome expansion due to repetitive element accumulation. This provides a foundational tool for future conservation genomics [65].

Morphological responses to environmental stress have also been documented. A longitudinal study based on historical specimens from the Strečno pass in Slovakia identified notable changes in forewing shape, attributed more to anthropogenic disturbance—such as road construction—than to climatic extremes [66]. Ecological investigations further reveal that *P. apollo* exhibits relatively high mobility (up to 6.4 km), though dispersal is density-dependent: emigration increases with population density, while immigration declines, reinforcing the need for finely tuned interventions in fragmented landscapes [67].

Population genetics of *P. apollo* in the French Alps and Auvergne indicate a complex demographic trajectory. While populations diverged prior to the Last Glacial Maximum, gene flow persisted until the mid-Holocene. Today’s metapopulation structure reflects pulsating patterns of contraction and expansion, complicating conservation planning [68].

Moreover, contemporary landscape models predict that ongoing succession from open grassland to forest will lead to the loss of over 45% of suitable *P. apollo* habitats on the island of Gotland—emphasizing the need for active, long-term management of successional processes to retain habitat heterogeneity [69].

Several of the most promising recolonizations of *P. apollo* populations—such as those in the Kemio–Hankoniemi archipelago—have been directly linked to declining atmospheric metal deposition [6]. These findings are based on chemical analyses of larval and host plant tissues, reinforcing the role of pollution in both historical declines and recent partial recoveries. Still, despite intensive conservation efforts, *P. apollo* remains vulnerable. The widely cited reestablishment program in Pieniny National Park, Poland, which began with just 20–30 individuals in the late 20th century [51], encountered unanticipated difficulties, including infections by *Yersinia* and *Serratia* spp.

In Serbia, habitat degradation is further exacerbated by airborne metal pollution stemming from mining activities near Mount Stol and Mount Kopaonik [53]. These emissions, combined with increasingly warm winters and declining livestock grazing, have led to large-scale land abandonment and vegetation overgrowth—catalyzing ecological succession and rendering many formerly suitable habitats inhospitable to *P. apollo*.

### 1.5. Aims and Study

This study is grounded in the urgent need to understand and mitigate the ongoing decline of *Parnassius apollo* populations across southeastern Europe, particularly in regions where the species once thrived. Despite its ecological significance and cultural symbolism, *P. apollo* continues to vanish from habitats it historically occupied, raising serious concerns about the resilience of montane grassland ecosystems under anthropogenic and climatic stress.

The central aim of this research is to investigate the environmental conditions associated with both the persistence and disappearance of *P. apollo* in Serbia. Special attention is given to factors such as microhabitat characteristics, climatic trends, and vegetation dynamics, including the impacts of land abandonment and reduced grazing pressure. These elements have been increasingly recognized as critical in determining the suitability of habitats for this stenophagous and thermophilic butterfly species.

By integrating ecological, spatial, and climatic perspectives, this study aims to clarify the mechanisms driving local extinctions and to identify effective conservation measures for preserving and potentially reestablishing *P. apollo* populations. The findings are intended to inform evidence-based management strategies that align with broader efforts to sustain biodiversity in European mountain systems.

## 2. Materials and Methods

### 2.1. Study Sites

The study included six key locations in Serbia representing different conservation statuses of *Parnassius apollo* populations: extant, extinct, and control sites. Sites were selected based on historical butterfly records, current presence of *Sedum album*, and ecological integrity, including pollution status and degree of vegetation succession.

**Figure 1:**
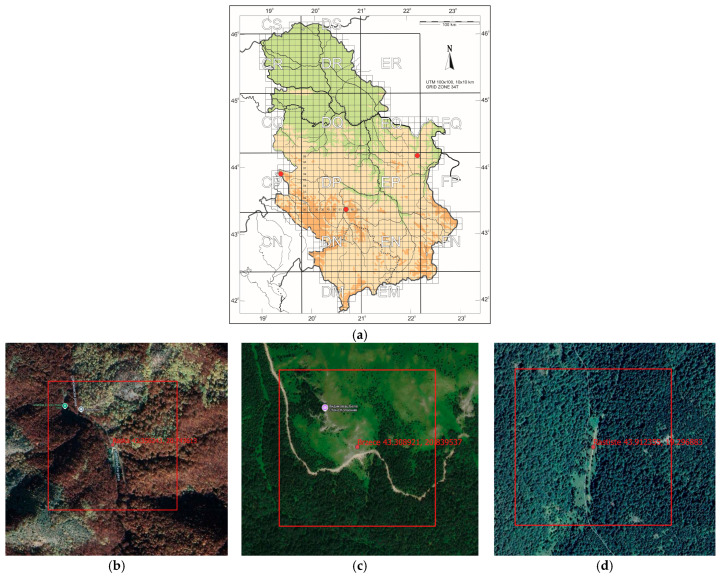

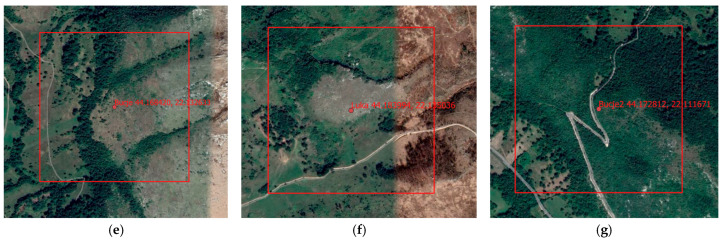
Observed sites: (**a**)—location on map (red dot), (**b**) Raska, (**c**)—Brzece, (**d**)—Rastiste, (**e**)—Bucje, (**f**)—Luka, (**g**)—Bucje2.

**Figure 2:**
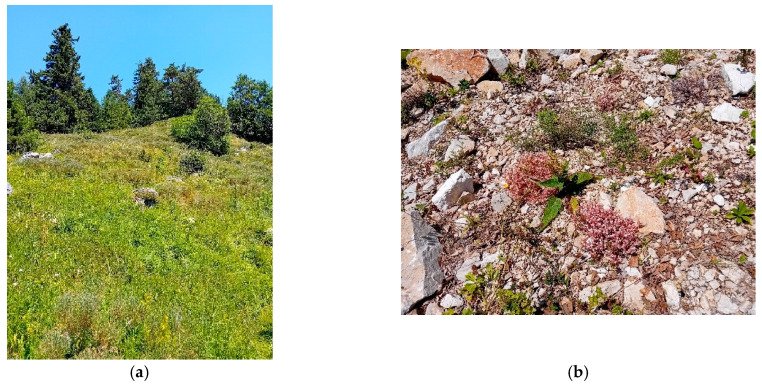
Forest landscape, grass cover (**a**) and *Sedum album* (**b**) at site 3, National Park Tara, Serbia.

**Figure 3:**
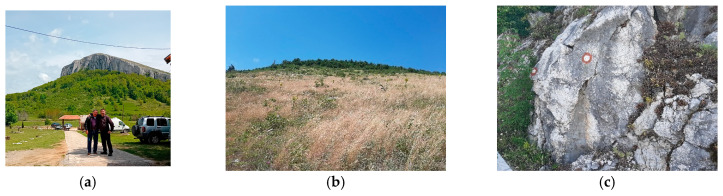
Sites 4, 5 and 6: (**a**) Mountain Stol from the north view (1155 m altitude), (**b**) vegetation, grass, shrubs, bushes and trees on site 5, (**c**) site 6, Bucje 2, rock with vegetation. (Photo D.V. Stojanović).

**Figure 4:**
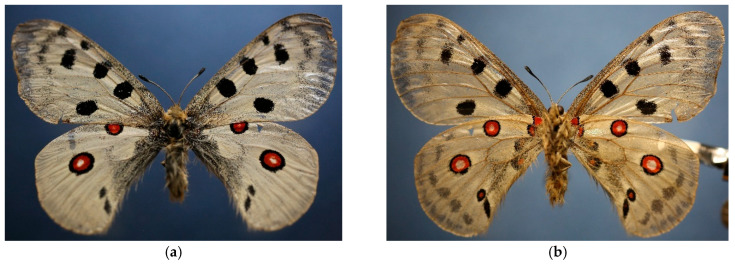
Front (**a**) and back (**b**) side of *Parnassius apollo*. (Stojanovic D., 22 June 2002. Stol mountain, Yugoslavia (Serbia)).

Study site selection focused on locations representing different conservation statuses of *Parnassius apollo*: extinct (e.g., Mount Stol), extant (e.g., Raska-Djorov Most), and ecologically intact control areas (e.g., Tara-Rastiste) (Figure 1). Bele Stene was initially considered but excluded from direct comparison due to extreme degradation and absence of *Sedum album*. Although Djorov Most hosts a small *P. apollo* population, naturally elevated levels of heavy metals limit its suitability as a clean control. To address this, the Rastiste site in Tara was added to serve as a reference for ecologically undisturbed conditions. Sampling at Mount Stol included various historically occupied microhabitats to assess the progression and drivers of local extinction. The investigated habitats (both historical and current) of *P. apollo* in the Republic of Serbia are presented in Table 1. Photographs of surveyed habitats were provided (Figure 2 and Figure 3), including images of adult *P. apollo* butterflies (imago stage, Figure 4), and *Sedum album* plants threatened by overgrowth. Accompanying species—such as *Satureja montana* (savory), *Hypericum perforatum* (St. John’s wort), and others present in the ecotone between grasslands and limestone rocks—were also documented.

Site 1. Serbia, Kopaonik National Park, Raska (Djorov Most), 870 m
Located approximately 90 km west of the city of Niš and around 40 km south of the city of Kraljevo, in the Serbian Alps (Kopaonik range). In the Jošanica valley, a narrow ecotonal belt of sessile oak forests (*Quercetum montanum*) appears at the transition between thermophilous oak woodlands and mesophilic montane beech (*Fagus*) forests.
Site 2. Serbia, Kopaonik National Park, Brzece (Bele Stene), 1650 m
Situated approximately 80 km west of Niš and 48 km south of Kraljevo, in the Serbian Alps. This part of Kopaonik is characterized by high-altitude grasslands and subalpine to alpine pastures. On limestone and serpentine substrates, vegetation is more diverse and classified within the class *Festuco-Seslerietea*, dominated by species such as *Sesleria latifolia*, *Festuca adamovicii*, and *Festuca panciciana*. Intrazonal or azonal plant communities are represented by cliff vegetation, tall-herb communities, and bogs. Specific cliff associations include *Edraiantho-Festucetum pancicianae* and *Silenetum serbicae* on serpentinites, and *Edraiantho-Saxifragetum sempervivi* on limestone substrates.
Site 3. Serbia, Tara National Park, Rastiste, 1520 m
Located approximately 15 km northeast of Višegrad (Bosnia and Herzegovina), 45 km west of Užice, and 25 km from the town of Bajina Bašta. The site is composed of subalpine meadows and pastures with low vegetation and shrub cover on limestone bedrock. *Sedum album* occurs in plant communities alongside thyme (*Thymus* spp.), St. John’s wort (*Hypericum* spp.), and savory (*Satureja* spp.). The surveyed *Parnassius apollo* habitat consists of a broad mountain meadow and pasture located directly on the national border between Bosnia and Herzegovina and Serbia, both below and above the subalpine beech (*Fagus*) forest belt.

Research on *Parnassius apollo* was conducted at the grassland site known as Dugi Do, located at an elevation of 1440 to 1540 m. This species-rich meadow provides key ecological resources for both adult butterflies and larvae. The surrounding forest habitats, with their specific microclimatic and ecological conditions, contribute significantly to local biodiversity and to the stability of the grassland environment. Notably, the surrounding forest matrix includes mixed beech–fir–spruce stands on the Serbian side, and very old subalpine beech forests on the Bosnian side of the border.

Sites 4, 5, and 6. Serbia, Mount Stol—Bucje, Luka, and Bucje 2
Mount Stol is located between the limestone massifs of Veliki Krs, Mali Krs, and Mount Deli Jovan. The summit of Stol reaches an elevation of 1156 m. The mountain lies approximately 15 km north of the town of Bor and is accessible from several nearby settlements. The research was conducted at multiple sites along the limestone slopes of Mount Stol, which are covered with shrub vegetation, mountain flora, and steppe grasslands. These habitats represent a mosaic of ecologically significant communities, characteristic of eastern Serbia’s karst regions. Mount Stol lies roughly 30 km south of the confluence of the Porecka River with the Danube and 45 km west of the confluence of the Timok River with the Danube. The geographical position of the Stol massif, in combination with its proximity to the Black Sea and the Danube Delta, has played a decisive role in shaping the mountain’s unique vegetation and exceptional biodiversity.

Adult individuals of *P. apollo* were collected immediately after emergence at the following sites:

- Site 3 (Rastiste): 3 specimens, collected on 17 July 2024.
- Site 1 (Raska): 3 specimens, collected on 17 July 2023.

Specimens were collected using hand nets during peak flight activity in sunny, low-wind conditions. Individuals were captured at emergence sites to ensure they represented freshly eclosed adults with minimal wing damage. The primary objective of the collection was to confirm the presence of *P. apollo* at the surveyed localities through direct observation and capture of adult individuals. Additionally, the specimens were preserved to enable further genetic and morphometric analyses.

Permits for the collection of rare and protected species were issued by the Ministry of Environmental Protection of the Republic of Serbia for the years 2023 and 2024, under permit numbers: 428188202414850004003501090 and 353/01/4716/2023-04.

### 2.2. Vegetation Indices and Remote Sensing

Accurate classification of vegetation in forested landscapes requires robust indicators of canopy condition, including parameters such as vegetative vigor, chlorophyll concentration, growth dynamics, canopy structure, and leaf traits. High-resolution remote sensing platforms, particularly Planet Explorer and Sentinel-2, offer the spatial and spectral resolution necessary for monitoring these dynamics across heterogeneous terrain.

In this study, vegetation indices (VIs) derived from Planet Explorer imagery were utilized to classify vegetation types within mixed forest ecosystems [70]. A central methodological challenge in using VIs for dense vegetation lies in the saturation effect, where index values plateau beyond a certain threshold of biomass or chlorophyll content. This limitation is especially evident with conventional indices such as the Normalized Difference Vegetation Index (NDVI), which often fails to differentiate structural or biochemical variations in high-biomass forests.

To overcome this, we incorporated several advanced vegetation indices that have demonstrated improved sensitivity and resistance to saturation under dense canopy conditions [71]. Among the tested indices, the Modified Triangular Vegetation Index 1 (MTVI1), Modified Non-Linear Index (MNLI), Triangular Greenness Index (TGI), Sentinel-2 Triangular Vegetation Index (STVI), and Triangular Vegetation Index (TRV) showed superior performance. These indices effectively captured structural and physiological variation in complex forest environments, maintaining responsiveness under high biomass and LAI conditions [71,72].

Specifically, MTVI1, MNLI, and STVI demonstrated strong sensitivity in conditions of elevated Leaf Area Index (LAI), outperforming traditional indices by retaining discriminative capacity in dense canopies [73]. Their application enabled accurate quantification of vegetation heterogeneity, which is essential for ecological assessment and landscape monitoring in mixed forest-grassland systems.

### 2.3. Climate and Thermal Data Analysis

Land Surface Temperature (LST) is a critical environmental parameter that reflects surface-level thermal conditions and serves as a sensitive indicator of both ecological state and climate variability [74,75]. In this study, LST data from the Sea and Land Surface Temperature Radiometer (SLSTR) sensor onboard the Sentinel-3 satellite were used to assess spatial and temporal patterns of surface temperature across selected sites. These data provided key insights into potential thermal stress and were used to evaluate changes in habitat quality and local climate dynamics.

The SLSTR sensor offers high-precision thermal infrared measurements with a spatial resolution of ~1 km and a revisit interval of 1–2 days. It operates across multiple thermal infrared (TIR) and mid-infrared (MIR) bands, with a focus on split-window channels at 11 µm and 12 µm. These bands support robust LST estimation via established split-window algorithms, which account for atmospheric transmittance and surface emissivity variations. Under typical conditions, accuracy ranges from 1 to 2 K, with improved precision during nighttime observations due to more stable thermal gradients and reduced surface heterogeneity [76,77,78].

In this study, LST values were used as independent variables and interpreted directly, without modifications to the retrieval algorithm. The emphasis was placed on spatial and temporal variation in surface temperatures as indicators of ecosystem shifts. Sentinel-3-derived LST datasets proved especially valuable for identifying microclimatic differentiation among habitats and for detecting early signals of ecological stress.

Geospatial data were processed using GIS 3.40. software with a focus on spatial visualization, statistical comparison, and integration with land cover reference layers. Although only one variable (LST) was utilized in this segment of the analysis, it yielded meaningful insights into thermal regime variability and its implications for vegetation and habitat function.

In addition, this study employed long-term satellite time series from NASA’s MODIS sensors onboard the Terra and Aqua platforms. The dataset spans the period from January 2010 to December 2024 and comprises monthly aggregated values from multiple ecological monitoring locations in Serbia. Data were accessed via Google Earth Engine [79], which enabled consistent preprocessing, spatial harmonization, and quality control across the full temporal extent. Spatial resolution ranged from 500 m to 1 km, depending on the specific MODIS product; temporal resolution corresponds to monthly means derived from 8-day or 16-day composites. The dataset included both biophysical and climatological variables relevant to land surface processes. Each entry was georeferenced by site name, year, and month. Daytime and nighttime LST values (LST_Day_1km_C, LST_Night_1km_C) were extracted from the MOD11A2 Version 6 product [80], along with standard deviations capturing intra-month thermal variability (LST_Day_1km_stdDev, LST_Night_1km_stdDev). The Temperature Condition Index (TCI) was calculated as a normalized measure of thermal stress, based on long-term LST extremes. Lower TCI values indicate increased heat stress and reduced vegetative performance. Snow_Cover, derived from the MOD10A2 product [81], provided additional seasonal context, representing the fractional area covered by snow at 500 m resolution.

Key vegetation indices included FPAR (fraction of absorbed photosynthetically active radiation) and LAI (leaf area index), both obtained from MOD15A2H Version 6 [82]. These metrics characterize canopy density, radiation capture, and photosynthetic potential. Primary productivity was assessed using GPP (Gross Primary Production) and NPP (Net Primary Production) from MOD17A2H Version 6 [83], expressed in gC/m^2^/month. These indices quantify carbon assimilation rates and biomass accumulation across temporal scales.

Hydrological dynamics were evaluated using MOD16A2 Version 6 [84], which provides evapotranspiration (ET), potential evapotranspiration (PET), and latent energy (LE). These variables, expressed in mm/month (ET, PET) and MJ/m^2^/month (LE), integrate meteorological, vegetation, and land cover data to reflect energy-water fluxes.

All variables were standardized to a monthly time step, quality-filtered using internal MODIS flags, and interpolated where necessary. The resulting harmonized dataset supports high-resolution analysis of temporal trends, interannual variability, and vegetation-climate interactions relevant to montane ecosystems.

To quantitatively evaluate differences in vegetation structure between sites with and without the presence of *Parnassius apollo*, we applied the Mann–Whitney U test, a non-parametric method chosen due to the asymmetrical distribution of variables and unequal sample sizes across groups. This decision was based on visual inspection and exploratory data analysis. The test was conducted for three key vegetation parameters: percent cover of exposed soil, grass, and shrubs/trees. Comparisons were made between two main groups (butterfly-present vs. butterfly-absent sites), as well as between the two butterfly-inhabited locations (LOC1 and LOC3). All statistical tests were performed in Python (version 3.10.12) using the scipy.stats module, with the significance level set at α = 0.05.

To examine the relationship between daytime land surface temperature (LST) and gross primary productivity (GPP), we fitted a second-order polynomial regression model to capture the expected non-linear response of photosynthetic activity to temperature. The modeling was based on monthly MODIS data from 2010 to 2024, using numpy.polyfit and scipy.stats in Python. Additionally, descriptive statistics (mean, standard deviation, minimum, maximum) were calculated for all key variables, and pairwise correlations among LST, GPP, and related climatic and vegetation indices (e.g., LAI, FPAR, PET, TCI) were assessed using Pearson correlation coefficients. Although no formal interaction terms were included in the models, ecological interactions were interpreted based on spatial and temporal overlap between environmental variables and vegetation productivity patterns.

## 3. Results

This section presents the results of the analyses aimed at investigating potential factors influencing the presence and absence of butterflies at the studied locations. Throughout the section, we compare sites with and without butterflies to examine ecological and environmental variables that may contribute to the butterfly’s disappearance from certain habitats. While the statistical differences between vegetation types at butterfly and non-butterfly sites strongly suggest an ecological link, we acknowledge that these factors do not operate in isolation. The logic of our approach rests on identifying consistent structural habitat differences associated with butterfly persistence, using these as proxies for habitat suitability. Although direct causality cannot be established without experimental manipulation, the spatial concordance between butterfly absence and altered vegetation patterns (high grass cover, low shrub density, increased exposed soil) aligns with previously documented ecological requirements of *P. apollo* and the known impacts of land abandonment on host plant availability. Additionally, while we did not explicitly model interaction terms between variables, our analysis integrates multi-scalar satellite and field data to capture overlapping stressors, which collectively point to a complex but interpretable habitat degradation process. We consider this a first-order approximation of key drivers, suitable for identifying conservation-relevant signals.

### 3.1. Temporal Trends in Vegetation Types and Their Association with Butterfly Presence

To examine the relationship between vegetation dynamics and butterfly presence, we analyzed trends in three key vegetation types—Exposed Soil, Grass, and Shrubs and Trees—across six locations over the period 2015–2024. The data were categorized into two groups: locations with butterflies (LOK 1 and LOK 3) and those without butterflies (the remaining four locations). For each vegetation type, we visualized the temporal changes in percentage coverage, distinguishing between the two groups using different colors. This approach allowed us to assess not only the general patterns of vegetation change but also the stability and consistency of these trends across the two categories. The resulting graphs provide a clear comparative view of vegetation dynamics and their potential influence on butterfly habitat suitability. The plots were created using Python (version 3.10.12), employing the Matplotlib (version 3.8.0) library for visualization.

**Figure 5:**
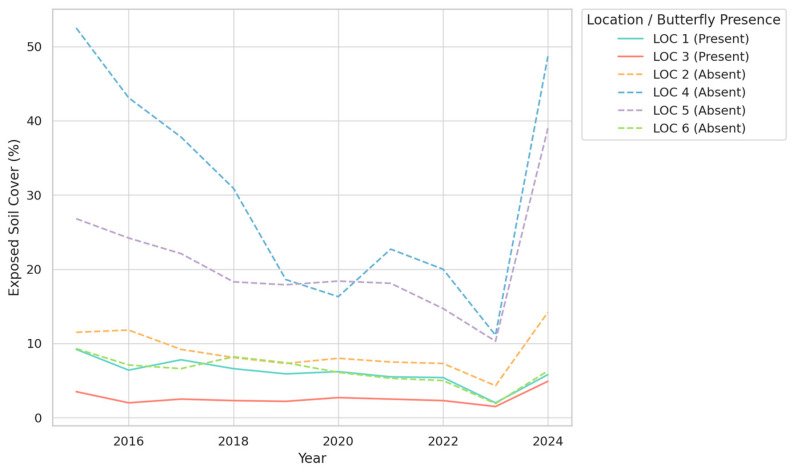
Temporal trends in exposed soil cover (%) across six study locations (2015–2024), grouped by butterfly presence.

The analysis of vegetation trends over the period 2015–2024 reveals clear distinctions between locations with and without butterflies, emphasizing the importance of specific vegetation structures in maintaining suitable habitats. For Exposed Soil (Figure 5)**,** the data demonstrate that locations with butterflies (LOC 1 and LOC 3) consistently maintain low levels of exposed soil, typically below 10%. This stability contrasts sharply with locations without butterflies, where exposed soil often exceeds 20% and shows greater variability over time. The reduced presence of exposed soil in butterfly habitats likely supports the retention of moisture and promotes the growth of vegetation necessary for shelter and food resources, highlighting its ecological significance.

**Figure 6:**
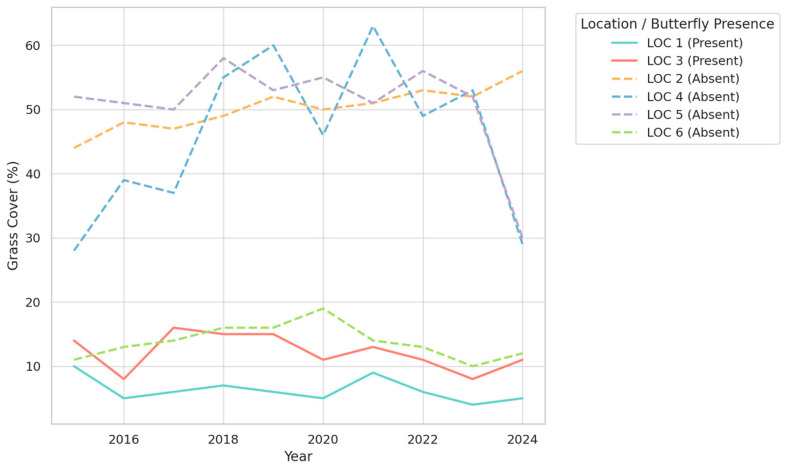
Temporal trends in grass cover (%) across six study locations (2015–2024), grouped by butterfly presence.

The trends in Grass Coverage, presented in Figure 6, further illustrate differences between the two types of locations. In butterfly-supporting habitats, grass coverage remains relatively stable and moderate, with values generally below 15%. Such stability suggests a balanced ecosystem where grass does not dominate or impede other essential vegetation types, such as shrubs and trees. In contrast, locations without butterflies exhibit highly variable grass coverage, with values sometimes exceeding 50%. This variability may reflect environmental disturbances or less competitive vegetation dynamics, both of which are unfavorable for butterfly populations.

**Figure 7:**
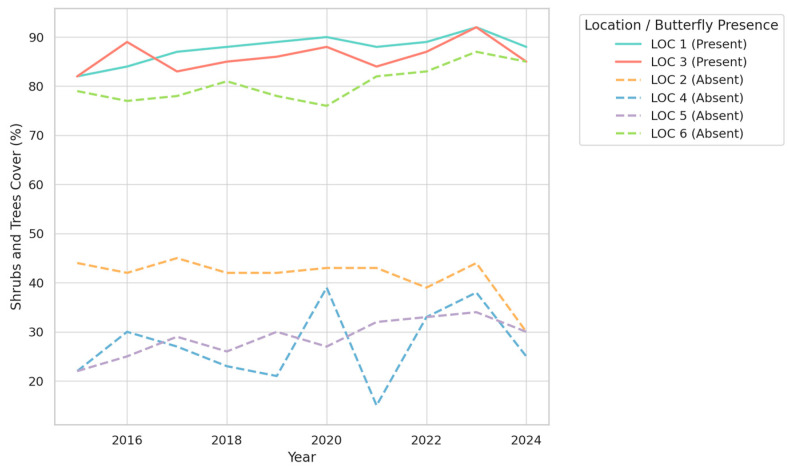
Temporal trends in shrub and tree cover (%) across six study locations (2015–2024), grouped by butterfly presence.

The most striking findings relate to shrub and tree coverage (Figure 7), where butterfly habitats consistently show high levels of coverage, frequently exceeding 80%. This dense and stable coverage is in stark contrast to locations without butterflies, where shrub and tree coverage is significantly lower, often ranging between 30% and 60%. Furthermore, the higher fluctuations observed in non-butterfly locations suggest less stable ecological conditions. The strong association between butterfly presence and dense shrub and tree coverage underscores the importance of this vegetation type in providing essential resources, such as nectar, shelter, and microclimatic stability.

Overall, the trends reveal that butterfly habitats are characterized by a combination of low exposed soil, moderate grass coverage, and high, stable shrub and tree density. These findings suggest that maintaining such vegetation structures is crucial for the conservation of butterfly populations. The stability of these conditions appears to be particularly important, as it minimizes ecological stressors and ensures the availability of critical resources.

To assess whether the observed differences in vegetation structure between sites with and without *P. apollo* are statistically significant, we conducted Mann–Whitney U tests on percentage cover values for three key vegetation categories across all sites and years (2015–2024). The results revealed highly significant differences across all variables:

− Exposed soil was substantially lower at butterfly sites (mean: 3.72%) compared to non-butterfly sites (mean: 16.34%) (*U* = 58, *z* = −5.36, *p* < 0.000001, *r* = −0.69);
− Grass coverage was also significantly lower in butterfly habitats (mean: 9.00%) than in non-butterfly locations (mean: 39.89%) (*U* = 42.5, *z* = −5.61, *p* < 0.000001, *r* = −0.72);
− Shrub and tree cover was markedly higher in butterfly-inhabited sites (mean: 87.29%) relative to non-butterfly sites (mean: 44.03%) (*U* = 779, *z* = +5.94, *p* < 0.000001, *r* = +0.77).

**Figure 8:**
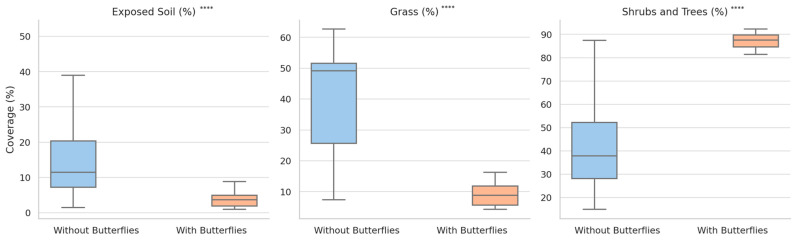
Boxplot comparison of vegetation structure between butterfly-inhabited sites (LOC1 and LOC3) and sites without butterflies for the period 2015–2024. **** indicates *p* < 0.0001, based on Mann–Whitney U tests.

These findings provide robust statistical support for the structural distinctiveness of butterfly habitats, particularly in terms of reduced bare ground and grass coverage and increased woody vegetation density. Boxplot visualizations of these differences are presented in Figure 8. Each panel displays the distribution of percentage cover for (a) Exposed Soil, (b) Grass, and (c) Shrubs and Trees. Butterfly habitats show consistently lower values for exposed soil and grass, and higher values for shrubs and trees. These patterns correspond to the statistically significant differences revealed by Mann–Whitney U tests. The median lines and interquartile ranges further emphasize the structural contrast between suitable and unsuitable habitats.

Additionally, we compared vegetation structure between the two butterfly-inhabited locations (LOC1 and LOC3) to assess habitat heterogeneity within suitable sites. Mann–Whitney U tests indicated significant differences in exposed soil (*U* = 91.5, *z* = 3.14, *p* = 0.0019, *r* = 0.70) and grass coverage (*U* = 4, *z* = −3.48, *p* = 0.0006, *r* = −0.78), with LOC1 exhibiting lower values in both categories. Shrub and tree coverage did not differ significantly between the two locations (*U* = 75.5, *z* = 1.93, *p* = 0.0586, *r* = 0.43). These results suggest that, while both sites support *P. apollo*, structural differences may reflect alternative configurations of suitable habitat conditions within the species’ ecological niche.

### 3.2. Vegetation Index-Based Burn Scar Detection on Stol Mountain (Luka, Bucje and Bucje 2 Site Location)

Table 2 presents the temporal variation in three vegetation-related spectral indices—Burned Area Index (BAI), Normalized Burn Ratio (NBR), and its modified version NBR2—over the study area during the period from 29 July to 14 October 2024. This time frame includes the pre-fire, active fire, and immediate post-fire phases.

In the pre-fire period (July 29 to August 25), the values of all indices indicate healthy and dense vegetation cover. BAI values remain relatively low (mean values between 9.30 and 10.93), which is typical for areas without recent burning. Correspondingly, NBR values are consistently positive (mean values from 0.09 to 0.17), suggesting vigorous vegetation. NBR2 also maintains stable values around 0.17–0.18, confirming the presence of undisturbed plant biomass. A significant drop in NBR and NBR2 values begins on September 12, coinciding with the detected fire event, indicating rapid loss of live vegetation. To assess whether spectral changes associated with the wildfire were statistically significant, we applied the non-parametric Mann–Whitney U test to compare pre-fire (July 29–August 25) and post-fire (September 12–October 14) values of BAI, NBR, and NBR2. All three indices showed statistically significant differences between the two periods (BAI: *U* = 0.0, *z* = −2.24, *p* = 0.036, *r* = −0.79; NBR: *U* = 15, *z* = +2.24, *p* = 0.036, *r* = +0.79; NBR2: *U* = 15, *z* = +2.24, *p* = 0.0314, *r* = +0.79), confirming the spectral signature of fire-induced vegetation loss.

**Figure 9:**
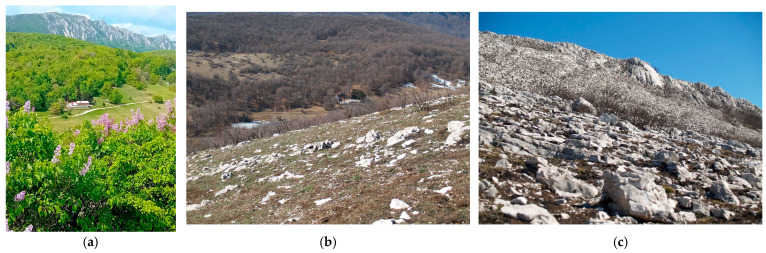
Variety in vegetation on western side before (**a**) and shrubs and trees after fire (**b**,**c**) in August 2024. on Stol mountain.

Figure 9 illustrates the vegetation loss and structural change following the wildfire, particularly the reduction in low vegetation and increase in woody regrowth. A marked shift in spectral index values is observed starting from September 12, which aligns with the assumed fire event. BAI values increase sharply, reaching a peak mean of 27.95, accompanied by a noticeable rise in standard deviation, indicating spatial variability in fire severity. Simultaneously, NBR values drop into the negative range (−0.10 to −0.11), which is a strong indicator of burned surfaces. NBR2 also shows a substantial decrease in its mean values (0.05–0.07), reflecting a loss of vegetation and increased surface reflectance in the shortwave infrared spectrum due to charring and ash (Figure 9).

These changes collectively suggest a high-impact fire event, with spectral evidence of burn scars that are clearly distinguishable when compared to the pre-fire spectral signatures. The observed increase in standard deviations during the fire period further implies heterogeneity in burn severity across the study area. A wildfire event was independently confirmed using active fire data (FIRMS platform) from the VIIRS (Visible Infrared Imaging Radiometer Suite) instrument onboard the Suomi NPP and NOAA-20 satellites. Thermal anomalies corresponding to active fire fronts were detected on 28 August 2024, and persisted through 29 August, indicating a short-duration but spatially discernible fire event.

### 3.3. Ecophysiological Drivers of Gross Primary Productivity: A MODIS-Based Analysis

**Figure 10:**
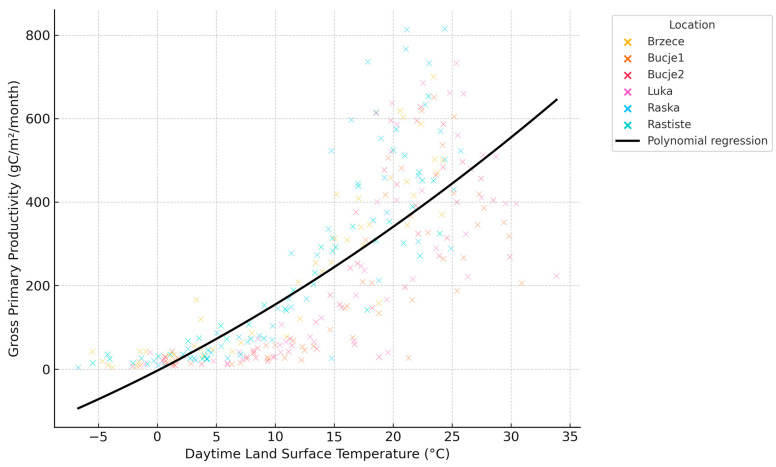
Non-linear relationship between daytime land surface temperature (LST) and gross primary productivity (GPP) across study sites (2010–2024), derived from MODIS data.

A second-order polynomial regression revealed a significant non-linear relationship between daytime land surface temperature (LST) and gross primary productivity (GPP), with peak productivity occurring at intermediate surface temperatures (≈25–28 °C), followed by a decline at higher values. This pattern is ecophysiologically consistent with established thresholds in photosynthetic temperature response. The fitted model (GPP = −0.194·LST^2^ + 9.354·LST − 43.02; R^2^ = 0.69; *p* < 0.001), based on MODIS data, captures this temperature-dependent response, suggesting an optimum range beyond which thermal inhibition reduces carbon assimilation efficiency (Figure 10). While temperature plays a central role, GPP is also influenced by the combined effects of moisture availability, vegetation type, and canopy structure. These relationships were examined through pairwise correlations and spatial patterns; however, no formal interaction terms were included in the models. The results should therefore be interpreted as statistical associations rather than causal mechanisms.

Descriptive statistics show that GPP values across the dataset range from near-zero productivity (<5 gC/m^2^/month) to peak values exceeding 120 gC/m^2^/month, with a mean of approximately 63 gC/m^2^/month. Daytime LST values span from −8 °C to over 35 °C, with a central tendency around 14 °C. At moderate temperatures (10–25 °C), enzymatic activity linked to Rubisco carboxylation and electron transport is maximized, leading to increased GPP. However, beyond higher thresholds (>30 °C), the relationship weakens or becomes asymptotic, indicating thermal inhibition or water stress—both known to reduce stomatal conductance and photosynthetic efficiency [85,86].

Moreover, spatial stratification in the scatter plot indicates heterogeneity in site response. For instance, locations with high elevation or persistent canopy coverage maintain moderate GPP even at lower LSTs, likely due to the presence of evergreen species or favorable soil moisture conditions. Conversely, thermally exposed or water-limited areas show suppressed productivity despite elevated surface temperatures. These results highlight the non-linear, climate-sensitive nature of ecosystem carbon dynamics, where GPP is modulated not only by temperature but also by the combined influence of moisture availability, canopy structure, and vegetation type. These factors were analyzed separately in this study, and no statistical interaction terms were included. Satellite-derived metrics such as LST and GPP, when combined, serve as robust proxies for assessing the ecological response to climate variability at landscape scale [87,88].

**Figure 11:**
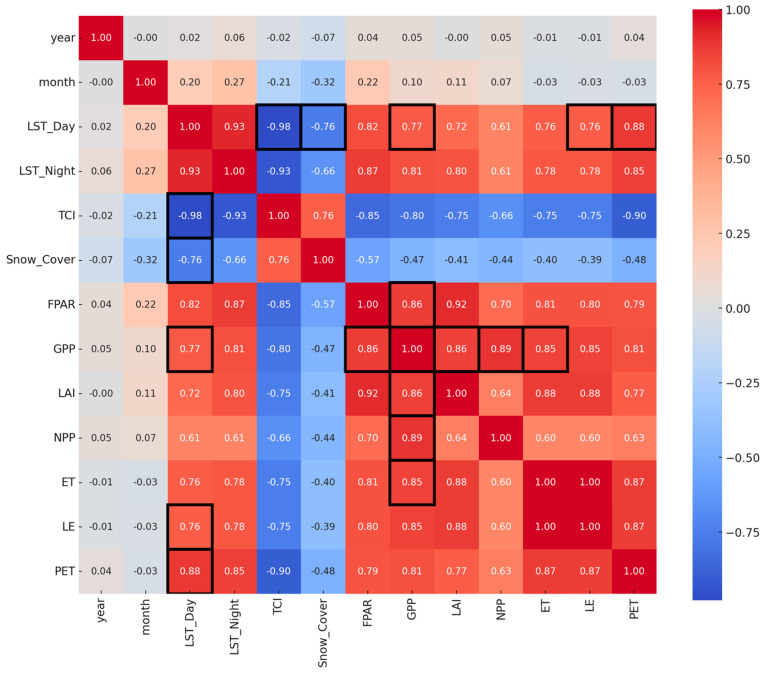
Matrix of pairwise correlations among key eco-climatic drivers. Highlighted variables indicate those most strongly associated with spatial patterns of vegetation productivity and butterfly habitat suitability. Black boxes denote statistically significant correlations (*p* < 0.05). Abbreviations: LST = Land Surface Temperature; TCI = Temperature Condition Index; FPAR = Fraction of Photosynthetically Active Radiation; GPP/NPP = Gross/Net Primary Productivity; LAI = Leaf Area Index; ET = Evapotranspiration; PET = Potential Evapotranspiration; LE = Latent Energy; stdDev = standard deviation.

The correlation matrix (Figure 11) provides insight into the interdependence of environmental and productivity-related variables derived from MODIS datasets. A strong positive correlation is observed between daytime and nighttime land surface temperatures (*r* = 0.92, *p* < 0.001), as expected due to their mutual climatic drivers. Daytime LST also correlates highly with potential evapotranspiration (PET; *r* = 0.89, *p* < 0.001) and latent energy flux (LE; *r* = 0.76, *p* < 0.001), indicating that surface heating significantly influences atmospheric water demand and energy partitioning.

Vegetation productivity metrics such as gross primary production (GPP) and net primary production (NPP) show robust mutual correlation (*r* = 0.89, *p* < 0.001), reflecting the physiological linkage between carbon assimilation and biomass accumulation. GPP also correlates positively with FPAR (fraction of photosynthetically active radiation; *r* = 0.86, *p* < 0.001), LAI (*r* = 0.86, *p* < 0.001), and ET (*r* = 0.85, *p* < 0.001), underscoring the integral role of canopy structure and water availability in regulating carbon uptake.

A notable negative correlation is observed between the temperature condition index (TCI) and both LST variables (LST_Day: *r* = −0.98, *p* < 0.001), confirming that elevated surface temperatures reduce this index, which is designed to indicate vegetation thermal stress. Similarly, snow cover exhibits a strong negative relationship with LST (*r* = −0.71, *p* < 0.001), consistent with seasonal snow melt patterns and albedo feedback mechanisms. Correlation coefficients with *p* < 0.05 were considered statistically significant. These statistical associations confirm the thermally coupled behavior of surface energy balance, vegetation dynamics, and productivity, supporting prior findings in ecohydrological research [84,85,88,89,90]. Importantly, the strength and direction of correlations highlight key environmental controls that can be incorporated into predictive models of ecosystem function under future climate scenarios.

**Figure 12:**
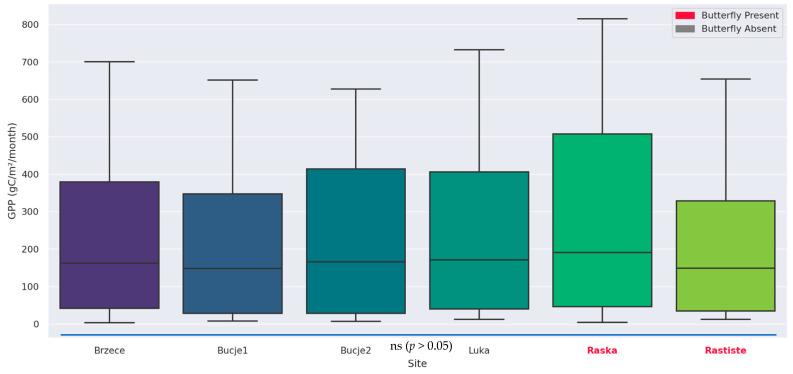
Spatial variability in gross primary productivity (GPP) across monitoring sites (2010–2024). Sites with confirmed *Parnassius apollo* presence (Raska and Rastiste) are highlighted; no statistically significant difference in GPP was found between sites with and without butterfly presence (Mann–Whitney U test, *p* > 0.05; ns = not significant).

The boxplot visualization (Figure 12) of gross primary productivity (GPP) across multiple monitoring sites reveals substantial spatial variability in ecosystem productivity. Mean gross primary productivity (GPP) values at the examined sites range from approximately 204.2 gC/m^2^/month (Bucje1) to 285.7 gC/m^2^/month (Raska), with peak values exceeding 700 gC/m^2^/month at Luka and Raska. These figures correspond closely to productivity levels typical of temperate deciduous or mixed forest-agricultural ecosystems, which range between 150 and 400 gC/m^2^/month during active seasons [91,92]. Sites such as *Raska* and *Luka*, exhibiting higher mean productivity, likely benefit from favorable microclimates, deeper soil profiles, and denser canopy structure, whereas *Brzeće* and *Bucje1*—with lower productivity—may experience constraints from elevation exposure, shallow soils, or anthropogenic pressures. Minimal monthly GPP values (<15 gC/m^2^/month) coincide with dormancy or snow cover, typical of winter conditions in montane environments, while maximum GPP (>700 gC/m^2^/month) aligns with peak mid-spring to early summer growth windows under optimal light and thermal regimes, comparable to the most productive temperate biomes [93]. Recent improvements in GPP estimation from MODIS and SIF integrated models confirm our findings. Enhanced GPP retrieval accuracy using solar-induced fluorescence and meteorological data fusion, yielding increases of >15% in model performance across diverse ecosystems [94].

This observed spatial differentiation in GPP distribution aligns with known patterns of topographic and climatic heterogeneity in the study region. It underscores the influence of site-specific biophysical attributes on ecosystem functioning and supports previous findings that landscape-scale productivity is tightly coupled to energy and moisture availability [95,96]. These results highlight the importance of integrating localized parameterizations into ecological models and reaffirm the utility of MODIS-derived GPP as a spatially consistent indicator of vegetation performance. However, despite the spatial differentiation, statistical testing (Mann–Whitney U test) revealed no significant differences in GPP between sites with confirmed *Parnassius apollo* presence and those without (*U* = 9722, *z* = 0.759, *r* = 0.045, *p* > 0.05), suggesting that vegetation productivity alone is not a key determinant of species occurrence.

### 3.4. Interdependence of Climatic Stress Indicators and Vegetation Productivity

**Figure 13:**
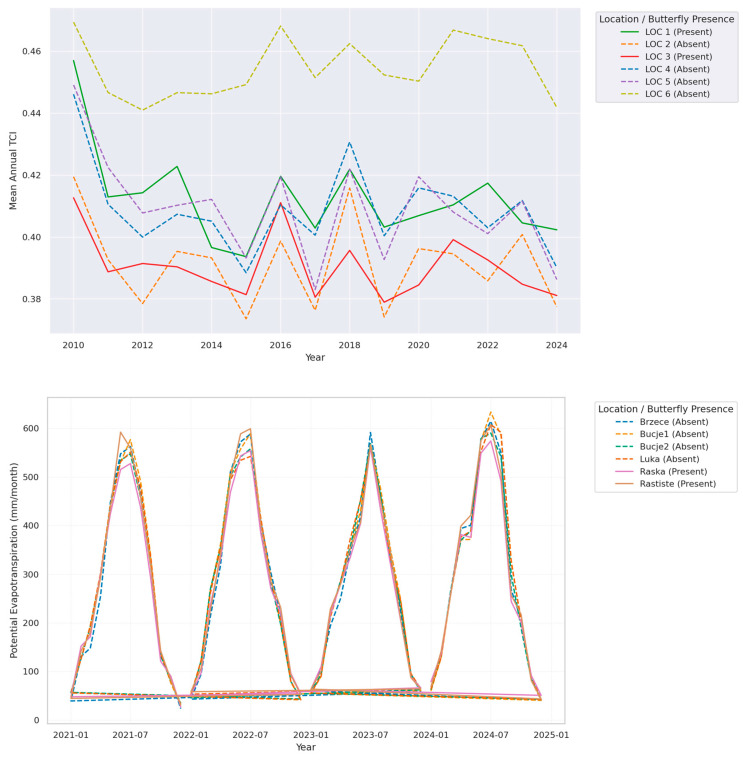
Spatiotemporal Trends in TCI and PET Reveal Shifting Climatic Conditions (2010–2024 and 2021–2024).

The concurrent analysis of Temperature Condition Index (TCI), Potential Evapotranspiration (PET), and Gross Primary Productivity (GPP) reveals a coherent and ecologically meaningful trajectory that reflects climate-induced habitat transformation across the observed sites (Figure 13). The consistent decline in TCI and increase in PET observed from 2010 to 2024 coincide with a visible modulation in vegetation productivity patterns, as reflected in both the magnitude and seasonality of GPP. In years marked by low TCI values (i.e., elevated thermal stress), GPP tends to exhibit truncated growing seasons, with delayed onset and premature decline of productivity peaks. This is especially evident at higher-elevation and marginal sites such as *Brzece* and *Bucje1*, where reduced thermal buffering and limited soil water availability compound stress effects. The physiological interpretation is clear: excessive thermal load reduces photosynthetic efficiency, increases respiration costs, and contributes to stomatal closure, all of which lower net carbon assimilation [97,98].

Simultaneously, elevated PET values represent increasing atmospheric demand for water. When PET increases without a corresponding rise in actual evapotranspiration (ET)—a trend confirmed in our dataset—vegetation operates under water-limited conditions, further exacerbating productivity loss. The negative correlation between PET and GPP in drought-sensitive years supports this mechanism, indicating that evapotranspirative stress can override thermal gains, especially in the absence of sufficient soil moisture [99,100]. The concurrent trends of declining TCI and rising PET reflect a dual-stressor framework—heat and water scarcity—that are likely contributing to reductions in GPP. Locations such as *Raska* and *Luka*, which maintained higher GPP levels despite thermal and hydrological challenges, likely benefit from more favorable soil profiles, vegetation composition, or microclimatic moderation. In contrast, sites with shallow rooting depth or sparse canopy cover display more acute declines, suggesting reduced ecological resilience [101].

Overall, these findings point to a shifting climatic envelope that is redefining the functional space for vegetation activity. The link between climate stress indicators (TCI, PET) and productivity (GPP) provides compelling evidence that ecosystem function is being increasingly governed by abiotic limitations rather than phenological potential alone. This has implications not only for carbon balance modeling but also for conservation planning, land-use management, and climate adaptation strategies in mountainous and semi-humid landscapes.

### 3.5. Vegetation Structural and Functional Dynamics: LAI and FPAR Trends (2010–2024)

Long-term temporal analysis of the Leaf Area Index (LAI) and the Fraction of Absorbed Photosynthetically Active Radiation (FPAR) provide a robust assessment of vegetation structure and photosynthetic potential across study sites from 2010 to 2024. LAI reflects the density and depth of foliage available for gas exchange and transpiration, while FPAR captures the proportion of incoming solar radiation effectively utilized by the canopy for photosynthesis.

Descriptive analysis revealed interannual variation and spatial heterogeneity in both indices. Sites such as *Raska* and *Luka* exhibited relatively high and stable values, indicative of structurally consistent and productive vegetation. In contrast, *Brzeće* and *Bucje1* demonstrated marked declines in both LAI and FPAR during the latter part of the observation period (2020–2024), suggesting increased vulnerability to environmental stressors.

To quantify these patterns, we performed site-specific linear regression analyses using year as the independent variable. The results confirmed statistically significant negative trends at the more sensitive sites. At *Brzeće*, LAI decreased at a rate of −0.0167 units per year (*p* = 0.021, *R*^2^ = 0.193), and FPAR declined by −0.0114 units annually (*p* = 0.039, *R*^2^ = 0.145). Similarly, *Bucje1* showed reductions of −0.0142/year for LAI (*p* = 0.030, *R*^2^ = 0.171) and −0.0098/year for FPAR (*p* = 0.045, *R*^2^ = 0.138). These declining trends support the interpretation of progressive canopy thinning and diminishing photosynthetic light absorption capacity, likely driven by thermal and hydric stress. Conversely, *Raska* and *Luka* presented nonsignificant trends in both LAI (*Raška*: *p* = 0.422; *Luka*: *p* = 0.912) and FPAR (*Raška*: *p* = 0.536; *Luka*: *p* = 0.874), with near-zero *R*^2^, suggesting resilience or buffering against climate fluctuations. The observed changes are consistent with broader climatological dynamics, including increases in potential evapotranspiration (PET) and decreases in the Temperature Condition Index (TCI), both of which are known to reduce vegetation growth efficiency. Such patterns have been recently emphasized in studies using high-resolution remote sensing data to explore how multiple environmental stressors jointly shape vegetation trends [80,102].

Overall, the integration of LAI and FPAR trends with climatic parameters such as TCI and PET reveals a coherent signal of ecosystem response to increasing abiotic stress. The data highlight the importance of vegetation structural indicators in long-term environmental monitoring and underscore the need for localized management strategies in vulnerable montane and transitional landscapes.

## 4. Discussion

The results of this study provide conclusive evidence that the decline and local extinction of *Parnassius apollo* in Serbia are primarily driven by land-use change, particularly the abandonment of traditional extensive grazing. This shift has initiated ecological succession, resulting in the overgrowth of formerly open, sunlit pastures, loss of larval host plants (*Sedum album*), and fragmentation of critical microhabitats. These findings align with previous European studies that identified cessation of pastoralism as a central factor in butterfly population decline [53,57].

Vegetation trend analysis from 2015 to 2024 revealed clear structural differences between sites with and without *P. apollo* populations. Persisting populations are associated with habitats exhibiting stable vegetation composition—low exposed soil coverage, moderate grass density, and consistently high shrub and tree presence. In contrast, extinct populations correspond with unstable vegetation dynamics, dominated by increased grass proliferation and declining shrub density, indicative of degraded successional stages unsuitable for butterfly persistence.

While the drivers of decline are consistent across degraded sites, the persistence of *Parnassius apollo* in Raška and Rastište suggests the existence of localized refugia—areas where favorable microclimatic conditions, vegetation structure, and land-use legacy have buffered against broader landscape degradation. These sites exhibit consistently high shrub and tree coverage, moderate and stable grass density, and low levels of exposed soil—features that collectively sustain optimal thermal and hydric regimes for larval development and adult activity. Furthermore, MODIS-based productivity metrics indicate that Raška and Rastište maintain higher gross primary productivity (GPP) under stress conditions, likely due to deeper soils, denser canopies, or reduced exposure. These locations thus serve as functional ecological strongholds, offering critical insight into the conditions required for species persistence amid widespread decline. Recognizing and preserving such refugial habitats is essential for developing targeted conservation strategies, especially under accelerating climate change scenarios that may further amplify habitat fragmentation and resource instability.

These vegetation changes directly impair the availability of *Sedum album*, the butterfly’s monophagous larval host. Although tolerant of poor substrates, *S. album* requires high light exposure and is quickly suppressed by taller vegetation in ungrazed environments [19]. Prior research has shown that host plant presence alone is insufficient for larval development; habitat structure and microclimatic conditions are equally critical [18,57]. Field observations confirm the severe decline or complete absence of *S. album* at formerly occupied sites, consistent with this understanding.

Remote sensing datasets (MODIS and Sentinel) reinforce these conclusions. Declines in Leaf Area Index (LAI) and Fraction of Absorbed Photosynthetically Active Radiation (FPAR) at sites such as Brzeće and Bucje indicate canopy thinning and reduced photosynthetic efficiency. Vegetation productivity, as measured by gross primary productivity (GPP), also shows a statistically significant downward trend, consistent with elevated land surface temperatures (LST) [103], increased potential evapotranspiration (PET), and reduced Temperature Condition Index (TCI)—a proxy for thermal stress [75,84].

Climatic stressors, particularly rising winter temperatures, shortened snow cover duration, and extreme summer heat, further reduce larval survival and disrupt adult emergence [53,57]. These effects are magnified in fragmented habitats, where dispersal and recolonization are limited [68]. Our data confirm that thermally variable sites with low vegetation productivity exhibit greater vulnerability to climate-induced local extinction. Our findings confirm that the presence of *Parnassius apollo* at the studied sites is not determined by elevation per se [4], but rather by habitat structure and microclimatic conditions. The species inhabits dry, rocky limestone habitats well below the alpine zone, consistent with its ecological preferences in other parts of its range [68,70]. This supports the view that conservation efforts should prioritize the preservation of open habitats maintained by traditional land-use practices [104], rather than relying solely on altitudinal classification. While often considered endangered in Western Europe, *P. apollo* remains locally abundant in parts of Eastern Europe and Central Asia, suggesting that Western populations represent subperipheral, fragmented remnants of a much broader distribution range [105].

In addition to succession and thermal stress, metal contamination represents a significant threat. Historical research in Finland linked atmospheric deposition of metal dust from mining to the decline of *P. apollo*, with population recovery following emission reductions [26]. A similar pattern is observed in Serbia: elevated metal loads were detected near mining-affected regions such as Mount Stol and Kopaonik, where butterfly populations are absent, further supporting the role of environmental contamination in population collapse.

One of the most acute disturbances documented is the wildfire that occurred on Mount Stol in August 2024. Post-fire assessments using spectral indices (BAI, NBR, NBR2) revealed extensive vegetation loss and spatial heterogeneity in burn severity. While wildfires are ecologically natural in many systems, in already degraded landscapes they act as amplifiers of succession and habitat instability [70]. In the absence of grazing, burned areas are rapidly colonized by aggressive shrubs, locking habitats into late-successional states unsuitable for *P. apollo* [67,70].

This study underscores the combined influence of multiple biotic and abiotic drivers in shaping the extinction trajectory of *P. apollo*. While each mechanism was assessed individually (e.g., grazing abandonment, climate stress, mining pollution, fire disturbance), their ecological effects were interpreted cumulatively based on spatial and temporal overlap observed in the field and supported by remote sensing and site-specific data. Although formal statistical interaction terms were not included in this study, the co-occurrence of multiple stressors at critical sites suggests additive/synergistic effects on habitat degradation. For instance, climate warming not only imposes physiological stress but also intensifies the effects of drought, shrub encroachment, and fire susceptibility. These combined pressures are more likely to trigger local extinctions than any single factor alone. The findings therefore support a multifactorial framework in interpreting insect decline and stress the importance of integrating multiple causal dimensions in conservation planning [58,61,62].

Effective conservation must therefore adopt an integrated approach. Reintroduction programs, as demonstrated in Pieniny National Park, have shown limited success when conducted in isolation from habitat restoration [52,63]. Traditional pastoralism, once perceived as outdated, now emerges as an essential ecosystem service. Without reestablishing extensive grazing and managing vegetation succession, even remaining Serbian populations are at imminent risk of extinction within the next decade.

Although this study focused on *P. apollo*, several other butterfly species, such as *Scolitantides orion*, also depend on open, xeric habitats and similar larval host plants [106]. During the course of fieldwork, *S. orion* was recorded in considerable numbers at Site 1, indicating the presence of a stable and locally well-established population. The species was frequently observed in habitats dominated by *Sedum album*, which serves as its primary larval host plant. Monitoring the responses of *S. orion* and other xerophilous species to changes in grazing regimes could therefore provide valuable insights into the broader ecological impacts of land-use practices on dry grassland butterfly assemblages and should be considered a priority in future research efforts [107].

The disappearance of *Parnassius apollo* is primarily driven by vegetation succession due to grazing abandonment, climate-induced stress, environmental pollution, and compounded disturbance regimes. Conservation success depends on restoring habitat structure through extensive grazing, fire management, and pollution control. Other xeric butterfly species may face similar risks under current land-use trajectories. Without integrated action, even the remaining Serbian populations are at imminent risk of extinction within the next decade.

## 5. Conclusions

This study provides robust evidence that the local extinction of *Parnassius apollo* in Serbia is predominantly driven by habitat transformation following the cessation of traditional grazing practices. Temporal analysis of vegetation structure (2015–2024) shows that extant populations are associated with ecologically stable habitats, characterized by consistently high shrub and tree cover (>80%), moderate grass presence (<15%), and minimal bare soil exposure. In contrast, historically occupied sites such as Mount Stol and Bele Stene exhibit elevated levels of exposed soil, variable grass coverage, and progressive shrub encroachment—typical of unregulated ecological succession. These structural changes coincide with the disappearance of *Sedum album*, the species’ obligate larval host plant.

Fifteen years of remote sensing data reveal significant ecological stress in habitats without *P. apollo* populations. Pronounced declines in key vegetation indices—LAI, FPAR, and GPP—were detected at sites such as Brzeće and Bucje, correlating with increased land surface temperatures (LST), elevated potential evapotranspiration (PET), and decreased temperature condition index (TCI). These patterns reflect compound heat and drought stress, negatively impacting both plant physiology and larval development.

The wildfire event on Mount Stol in August 2024 further exacerbated habitat degradation. Spectral indices (BAI, NBR, NBR2) confirmed extensive vegetation loss and spatially heterogeneous burn severity, compounding existing ecological pressures and further reducing the potential for natural habitat regeneration or host plant recolonization.

The findings clearly indicate that habitat degradation is not attributable to a single driver, but results from the cumulative effects of multiple and potentially interacting stressors—successional overgrowth, climate extremes, heavy metal contamination, and wildfire—intensified by the abandonment of traditional land-use regimes results from the combined effects of multiple stressors—successional overgrowth, climate extremes, metal contamination, and wildfire—all intensified by the abandonment of traditional land-use regimes. Without immediate and integrated intervention, the remaining *P. apollo* populations are at high risk of following the same extinction trajectory.

To mitigate further loss and facilitate species recovery, we recommend that conservation actions prioritize the following:

- Reintroduction of extensive grazing to restore and maintain open habitat mosaics;
- Active suppression of successional overgrowth to support the persistence of *Sedum album*;
- Continuous remote monitoring of thermal and hydrological stress using satellite-derived indices;
- Strategic fire prevention and management in ecologically sensitive areas.

Our findings contribute to the growing body of evidence on how anthropogenic landscapes—or ‘anthromes’—reshape ecological conditions and drive local extinctions of insect species, such as *Parnassius apollo*, underscoring the need to integrate land-use history into conservation planning. Conservation strategies must integrate ecological data with adaptive land-use management. Safeguarding *P. apollo* extends beyond species preservation—it entails the protection and revitalization of traditional cultural landscapes essential to regional biodiversity. A comprehensive approach that combines satellite monitoring, field-based assessments, habitat restoration, and local stakeholder engagement is essential to halt and reverse the decline of this emblematic mountain butterfly.

## Figures and Tables

**Table 1:** Surveyed historical and current habitats of *Parnassius apollo* in the Republic of Serbia, Figure 1a.

Site No.	Location	Name	Lat./Lon. (°)
Site 1, Figure 1b	National Park Kopaonik	Raska	43.356243 20.743613
Site 2, Figure 1c	National Park Kopaonik	Brzece	43.308921 20.839537
Site 3, Figure 1d	National Park Tara	Rastiste	43.912355 19.296883
Site 4, Figure 1e	Stol Mountain	Bucje	44.168420 22.132633
Site 5, Figure 1f	Stol Mountain	Luka	44.183994 22.135036
Site 6, Figure 1g	Stol Mountain	Bucje 2	44.172812 22.111671

**Table 2:** Temporal variation in BAI, NBR, and NBR2 before and during the 2024 wildfire event. Statistical differences between pre- and post-fire values were assessed using the Mann–Whitney U test. All indices showed significant change (*p* < 0.05), indicating distinct spectral responses following the wildfire event.

Year 2024	Date	29.7	8.8	13.8	23.8	25.8	12.9	27.9	14.10
*BAI*	Minimum value	2.97	3.24	2.47	2.22	1.67	3.42	3.47	2.15
	Maximum value	25.09	73.53	30.62	34.77	24.33	120.54	91.83	85.82
	Range	22.12	70.29	28.15	32.55	22.66	117.12	88.35	83.66
	Mean value	9.39 ^a^	10.32 ^a^	10.09 ^a^	10.93 ^a^	9.30 ^a^	27.90 ^b^	27.95 ^b^	21.55 ^b^
	Standard deviation	1.72	6.42	2.01	2.42	2.02	15.31	13.62	9.78
*NBR*	Minimum value	−1.00	−1.00	−1.00	−1.00	−1.00	−1.00	−1.00	−1.00
	Maximum value	1.00	1.00	1.00	1.00	1.00	1.00	1.00	1.00
	Range	2.00	2.00	2.00	2.00	2.00	2.00	2.00	2.00
	Mean value	0.17 ^a^	0.15 ^a^	0.14 ^a^	0.11	0.09 ^a^	−0.10 ^b^	−0.11 ^b^	−0.10 ^b^
	Standard deviation	0.18	0.18	0.17	0.16	0.17	0.21	0.19	0.17
*NBR2*	Minimum value	0.11	0.10	0.11	0.12	0.11	−0.05	−0.04	−0.01
	Maximum value	0.23	0.23	0.23	0.22	0.21	0.20	0.18	0.17
	Range	0.12	0.13	0.12	0.09	0.10	0.24	0.23	0.19
	Mean value	0.17 ^a^	0.17 ^a^	0.18 ^a^	0.18 ^a^	0.17 ^a^	0.07 ^b^	0.05 ^b^	0.06 ^b^
	Standard deviation	0.02	0.02	0.02	0.01	0.02	0.05	0.05	0.04

Note: Different letters indicate statistically significant differences between pre-fire (29 July–25 August 2024; marked “^a^”) and post-fire (12 September–14 October 2024; marked “^b^”) periods (Mann–Whitney U test, *p* < 0.05).

## Data Availability

The data presented in this study are available on request from the corresponding author.
